# Variational attenuation correction in two-view confocal microscopy

**DOI:** 10.1186/1471-2105-14-366

**Published:** 2013-12-18

**Authors:** Thorsten Schmidt, Jasmin Dürr, Margret Keuper, Thomas Blein, Klaus Palme, Olaf Ronneberger

**Affiliations:** 1 Department of Computer Science, Albert-Ludwigs-Universität, Georges-Köhler-Allee Geb. 52, 79110 Freiburg, Germany; 2 Institute of Biology II, Albert-Ludwigs-Universität, Schänzlestr. 1, 79104 Freiburg, Germany; 3 Current address: Institut Jean-Pierre Bourgin, Unité Mixte de Recherche 1318, Institut National de la Recherche Agronomique-AgroParisTech, Bâtiment 2, INRA Centre de Versailles-Grignon, 78026 Versailles Cedex, France; 4 BIOSS, Centre for Biological Signalling Studies, Albert-Ludwigs-Universität, Albertstr. 19, 79104 Freiburg, Germany; 5 FRIAS, Freiburg Center for Advanced Studies, Albert-Ludwigs-Universität Freiburg, Albertstr. 19, 79104 Freiburg, Germany; 6 FRISYS, Faculty for Biology, Albert-Ludwigs-Universität Freiburg, Albertstr. 19, 79104 Freiburg, Germany

**Keywords:** Attenuation correction, Absorption, Confocal microscopy, Image restoration, Calculus of variations

## Abstract

**Background:**

Absorption and refraction induced signal attenuation can seriously hinder the extraction of quantitative information from confocal microscopic data. This signal attenuation can be estimated and corrected by algorithms that use physical image formation models. Especially in thick heterogeneous samples, current single view based models are unable to solve the underdetermined problem of estimating the attenuation-free intensities.

**Results:**

We present a variational approach to estimate both, the real intensities and the spatially variant attenuation from two views of the same sample from opposite sides. Assuming noise-free measurements throughout the whole volume and pure absorption, this would in theory allow a perfect reconstruction without further assumptions. To cope with real world data, our approach respects photon noise, estimates apparent bleaching between the two recordings, and constrains the attenuation field to be smooth and sparse to avoid spurious attenuation estimates in regions lacking valid measurements.

**Conclusions:**

We quantify the reconstruction quality on simulated data and compare it to the state-of-the art two-view approach and commonly used one-factor-per-slice approaches like the exponential decay model. Additionally we show its real-world applicability on model organisms from zoology (zebrafish) and botany (Arabidopsis). The results from these experiments show that the proposed approach improves the quantification of confocal microscopic data of thick specimen.

## Background

Confocal microscopy has become a standard technique to record and localize fluorescent marker molecules within the 3-D context of organs and whole organisms on sub-cellular resolution. The confocal principle minimizes the blur introduced by the point spread function of the optics. However, signal degradations introduced by scattering and absorption within the inhomogeneous tissue still hamper many automatic image analysis steps like detection, registration, segmentation, or co-localization.

Light attenuation is a result of photon loss along the excitation and emission light paths. Photons get lost due to absorption, where the photons are converted to thermal energy, or due to scattering, where the photons leave the ray passing through the pinhole. Both effects result in a multiplicative reduction of the number of photons by a local tissue specific factor, and can therefore be modeled by the Beer-Lambert’s law. The opposite effect, an intensity increase, is caused by scattered photons that hit the pinhole by chance. In most tissues this second effect is small compared to the photon loss and its exact simulation would require an immense computational effort. Therefore we model only photon loss using attenuation coefficients accounting for both local absorption and scattering throughout the article.

Attenuation correction requires to estimate two quantities at each recording position, the local attenuation coefficient and the true underlying intensity. Solving for both quantities without further assumptions would require two noise-free measurements per recording position. However in most real-world applications only sparse measurements at the fluorescently marked structures are available (especially when imaging whole organs or organisms). Additionally the measured signal is distorted by Poisson distributed photon noise and Gaussian distributed read-out noise.

Single view approaches try to estimate both quantities from one recording that provides only one measurement per recording position. This requires strong prior assumptions to constrain the solution space. A common approach is to assume that the attenuation is dominated by aberrations introduced by a mismatch in immersion and embedding media [1,2]. In the resulting models, local attenuation effects are neglected or constant absorption throughout the cuboid-shaped recording volume is assumed resulting in an exponential decay with imaging depth [3]. Other approaches estimate the attenuation from the per-slice intensity statistics. The overall intensity distribution is adapted towards a reference maximizing the overall coherence [4,5].

**Figure 1:**
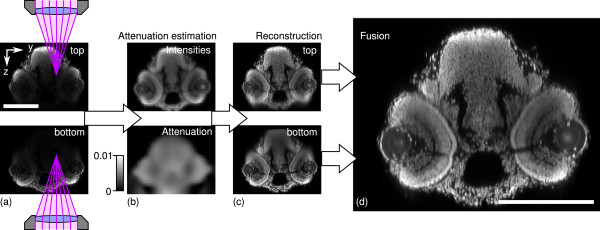
**The attenuation correction work flow.(a)** yz-sections of raw confocal stacks from two views (top/bottom). **(b)** Estimated real intensities and attenuation coefficients in the lower processing resolution. **(c)** Independently reconstructed intensities (high resolution) of the top- and bottom-views. **(d)** Final result after fusion of the reconstructed views. Scale bars indicate 200 *μ*m. Shown intensities are clipped to the [ 0,500] range.

One way of theoretically getting sufficiently many measurements to solve the problem is to record the sample from different angles (e.g. two views from opposite sides, see Figure 1). In [6] this has been done to increase the signal to noise ratio (SNR) of the reconstructed volume. The authors discuss, that previous approaches are only applicable given homogeneously distributed markers throughout the sample which is hardly the case. They propose instead to directly relate the absorption to the fluorophore distribution that can be observed. In [7], we go even one step further and assume no relationship between attenuation and marker, since only in rare cases all absorbing material is also fluorescently marked. The confocal image formation [7,8] allows to recover attenuation in not fluorescently marked areas as long as they cast “shadows” through the sample along the excitation and emission cones of the different views. Only in regions where the light hits no fluorophores at all or in the case of full absorption an estimation is impossible.

The multi-view approach can be applied to a wide range of data from biology and medicine. To underline this claim, we reconstruct the recordings of 500 *μ*m thick zebrafish embryos, and Arabidopsis root tips. Two-view recording of tissue sections was already demonstrated in [6].

### Contributions

**Figure 2:**
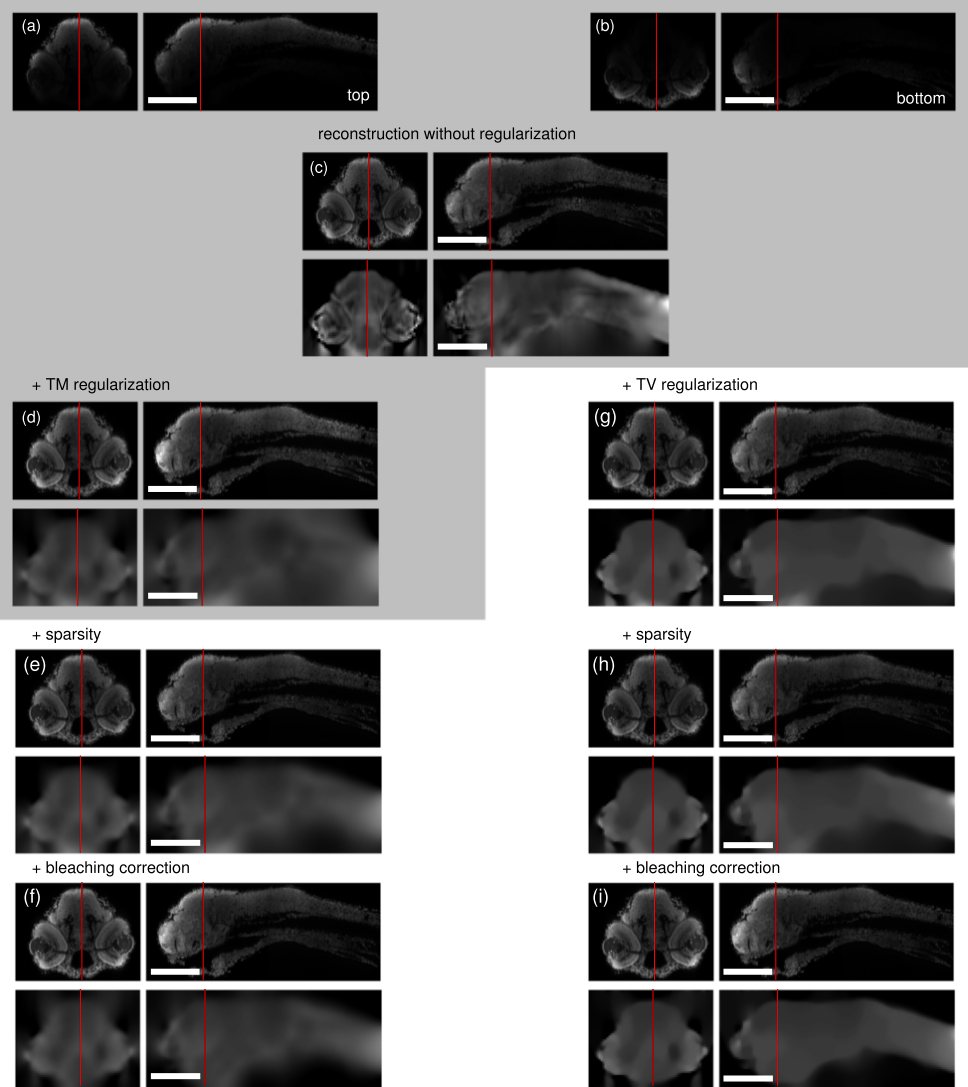
**Overview over the extensions to **[7]**.(a-b)**: yz- and xz-sections of raw confocal stacks from two views (top/bottom); **(c)**: optimal reconstruction without regularization; left: **(d)** with Tikhonov Miller regularization (*λ*=10^7^), **(e)** with sparsity (*μ*=1000), **(f)** with bleaching correction (est. *β*=0.64); right: **(g)** with total variation regularization (*λ*=10^4^), **(h)** with sparsity (*μ*=1000), **(i)** with bleaching correction (est. *β*=0.63). Parts in the gray block are already implemented in [7]. Scale bars: 200 *μ*m.

This work is a significant extension to the attenuation correction presented in [7] extending the ideas and the evaluation presented in [9]. First, we use an elastic registration to align the two views which allows embeddings in viscous media without mechanical fixation. Second, we reformulate the image formation model to cope with the photon noise apparent in confocal microscopy and photo bleaching which cannot be avoided when recording the same sample multiple times. Third, we examine the effects of different priors (Tikhonov-Miller, total variation, and sparsity) on the attenuation field, and finally, we ensure the constraints on the variables (attenuation coefficients must be positive) directly in the optimizer. The effects of the different extensions are illustrated in Figure 2.

## Methods

### Image formation model

We use the image formation model and algorithms from [7] to simulate a confocal microscope with ideal point spread function. Optimal reconstruction quality using this model requires that the refractive indices of immersion and embedding medium match the specification of the microscope lens. They should be adapted to the average refractive index of the imaged specimen. The signal *F*_*i*_(**x**) for direction *i* (*i*=1: top, *i*=2: bottom) captured by the photo multiplier is modeled as the integral over a cone shaped bundle of rays originating at the recording position **x**∈*ℝ*^3^. Each ray is attenuated by the attenuation coefficients along its path so that

(1) $$F_{i}\left(x\right)=\beta_{i}I\left(x\right)\cdot {\left(\int_{S}s_{i}\left(r\right)\cdot e^{-\int_{0}^{\infty }\alpha \left(x+\ell r\right)\;d\ell }\;dr\right)}^{2}\;,$$

where $\alpha :{\mathbb{R}}^{3}⊃\Omega \to {\mathbb{R}}_{\geq 0}$ are the spatially variant attenuation coefficients. *s*_*i*_:*S*→{0,1} are the cone sensitivity functions for both directions defined over the unit sphere *S*. *s*_*i*_(**r**) is one for all ray directions within the cone and zero otherwise. $I:\Omega \to {\mathbb{R}}_{\geq 0}$ denotes the attenuation free intensities. The factors *β*_*i*_∈*ℝ*_+_ can be used to additionally scale all intensities of the recordings. We use it to model photo bleaching induced signal attenuation in the second recording. Only the focused beam leads to significant bleaching since the excitation energy drops quadratically with the distance to the focal point. The assumption of constant bleaching for the whole volume is a zero-order approximation for the true bleaching function which is non-linear and specific for the marker-molecules used. We fix *β*_1_:=1 and optimize *β*_2_ alongside with the real intensities *I* and the attenuation coefficients *α* as described in the upcoming section.

### Energy formulation

We want to maximize the posterior probability for the attenuation coefficients *α*, the attenuation-free intensities *I*, and the factor *β*_2_ given the observed intensities of the two recorded data volumes *I*_1_ and *I*_2_.

According to Bayes’ rule we get

(2) $$\begin{array}{lcr} \left(\alpha^{*},I^{*},\beta_{2}^{*}\right) & = & \arg\underset{\alpha ,I,\beta_{2}}{\max}\frac{P\left(I_{1},I_{2}|\alpha ,I,\beta_{2}\right)P(\alpha ,I,\beta_{2})}{P(I_{1},I_{2})}\\ & = & \arg\underset{\alpha ,I,\beta_{2}}{\max}P\left(I_{1},I_{2}|\alpha ,I,\beta_{2}\right)P(\alpha ,I,\beta_{2})\;. \end{array}$$

The prior *P*(*I*_1_,*I*_2_) of the recorded images is independent of the attenuation, the true intensities, and the bleaching, therefore it could be dropped from the equation.

We have no prior knowledge about the expected intensities but want the attenuation coefficients to vary smoothly within local neighborhoods. Additionally we prefer solutions with zero attenuation estimates in areas of insufficient data. This can be modeled in the prior probability as

(3) $$P\left(\alpha \right)∼e^{-\lambda \underset{\Omega }{\int }\psi \left({∥\nabla \alpha ∥}^{2}\right)\;dx}\cdot e^{-\mu \underset{\Omega }{\int }\sqrt{\alpha^{2}\left(x\right)+\varepsilon_{\text{sp}}^{2}}\;dx}\;,$$

where $\Omega \subset {\mathbb{R}}^{3}$ is the recorded volume, *λ*,*μ*∈*ℝ*_≥0_ are weighting factors and *ε*_sp_∈*ℝ*_+_ is a small constant which is added for reasons of numerical stability. The loss function ψ:ℝ→ℝ is either the identity function, leading to quadratic regularization (Tikhonov Miller (TM)) or approximates the total variation regularization (TV) when set to $\psi_{\text{TV}}\left(x^{2}\right):=\sqrt{x^{2}+\varepsilon_{\text{TV}}^{2}}$ with *ε*_TV_∈*ℝ*_+_ being a small constant. This approximation is closely related to the Huber norm. During the experiments we set *ε*_sp_=*ε*_TV_=10^−10^.

We assume, that for all discrete recording positions **x** the observed attenuation coefficients and intensities are independent (except for their explicitly modeled common dependency on *α*, *I*, and *β*_2_) resulting in

(4) $$\;\left(\alpha^{*},I^{*},\beta_{2}^{*}\right)=\arg\underset{\alpha ,I,\beta_{2}}{\max}P\left(\alpha \right)\underset{x\in \Omega^{\prime }}{\prod }\overset{2}{\underset{i=1}{\prod }}P\left(I_{i}\left(x\right)|\alpha ,I,\beta_{2}\right)\;,$$

where *Ω*^′^ is the discretized recorded volume.

The noise in the image intensities is dominated by the Poisson distributed shot noise due to the quantum nature of light but the measured intensities also contain additive Gaussian distributed read-out noise. The resulting noise model is the convolution of a Poisson process (scaled by a constant factor *m*∈*ℝ*_+_ and offset *b*∈*ℝ*) with a zero mean Gaussian distribution with standard deviation *σ*∈*ℝ*_+_. We eliminate the offset *b* by subtracting the intensity at the histogram peak from each recording prior to further processing. For reasons of computational efficiency, we approximate the Poisson process by a Gaussian process with variable variance (the variance is proportional to the mean of the distribution). We further assume, that the measured intensities are a good estimator of this mean value. At each position **x**∈*Ω* this leads to a convolution of two Gaussian distributions which results in the combined Gaussian distribution

(5) $$\begin{array}{lll} \;P\left(I_{i}\left(x\right)|\alpha ,I,\beta_{2}\right)\approx & \frac{1}{\sqrt{2\pi m^{2}I_{i}\left(x\right)}}e^{-\frac{{\left(I_{i}\left(x\right)-\beta_{i}F_{i}\left(x\right)\right)}^{2}}{2m^{2}I_{i}\left(x\right)}}\; & \;\\ & *\frac{1}{\sqrt{2\pi \sigma^{2}}}e^{-\frac{{\left(I_{i}\left(x\right)-\beta_{i}F_{i}\left(x\right)\right)}^{2}}{2\sigma^{2}}}\; & \;\\ & =\frac{1}{\sqrt{2\pi \left(m^{2}I_{i}\left(x\right)+\sigma^{2}\right)}}e^{-\frac{{\left(I_{i}\left(x\right)-\beta_{i}F_{i}\left(x\right)\right)}^{2}}{2\left(m^{2}I_{i}\left(x\right)+\sigma^{2}\right)}}\;.\; \end{array}$$

Note, that for *m*=0 and *σ*=1 the new model coincides with the pure Gaussian model presented in [7]. The actual value for the Poisson scaling *m* (the number of collected photons per intensity level) and the standard deviation *σ* of the Gaussian noise can be estimated during a microscope calibration phase. If they are unknown, one of them can be fixed to an arbitrary value (we always fixed *σ*=1), and the other one can be adjusted to qualitatively obtain the optimum result. If additional sample information is available, e.g. the recordings consist of large homogeneous regions of different intensities, one can also try to estimate the parameters from the images themselves as done in [10]. However, for biological samples this is rarely the case.

The final energy formulation is obtained by replacing the maximization of the probability by a minimization of its negative logarithm

(6) $$\begin{array}{ll} \underset{\alpha ,I,\beta_{2}}{\min}E\left(\alpha ,I,\beta_{2}\right) & :=\underset{\alpha ,I,\beta_{2}}{\min}\underset{E_{\text{data}}}{\underset{︸}{\overset{2}{\underset{i=1}{\sum }}\underset{\Omega }{\int }\frac{{\left(I_{i}\left(x\right)-F_{i}\left(x\right)\right)}^{2}}{m^{2}I_{i}\left(x\right)+\sigma^{2}}\;dx}}\\ & \;+\lambda \underset{E_{\text{smooth}}}{\underset{︸}{\underset{\Omega }{\int }\psi \left({∥\nabla \alpha \left(x\right)∥}^{2}\right)\;dx}}\\ & \;+\mu \underset{E_{\text{sparse}}}{\underset{︸}{\underset{\Omega }{\int }\sqrt{\alpha^{2}\left(x\right)+\varepsilon_{\text{sp}}^{2}}\;dx}}\\ \text{respect to}\;\;\;\; & \beta_{2}>0\;\wedge \;\forall x\in \Omega :\alpha \left(x\right)\geq 0\;. \end{array}$$

To simplify the notation, we introduce the shorthands

$$\begin{array}{lcr} T_{r}\left[\alpha \right]\left(x\right) & := & e^{-\int_{0}^{\infty }\alpha \left(x+\ell r\right)\;d\ell }\\ C_{i}\left[\alpha \right]\left(x\right) & := & \underset{S}{\int }s_{i}\left(r\right)\cdot T_{r}\left[\alpha \right]\left(x\right)\;dr\\ F_{i}\left[\alpha ,I,\beta_{2}\right]\left(x\right) & = & \beta_{i}I\left(x\right)C_{i}^{2}\left[\alpha \right]\left(x\right)\\ D_{i}\left[\alpha ,I,\beta_{2}\right]\left(x\right) & := & I_{i}\left(x\right)-F_{i}\left[\alpha ,I,\beta_{2}\right]\left(x\right) \end{array}$$

where *T*_**r**_ is the attenuation along the ray with direction **r**, *C*_*i*_ is the cone transmission for recording direction *i*, *F*_*i*_ are the simulated intensities, and *D*_*i*_ are the differences between the recordings and simulations. Variables in square brackets indicate dependencies on the corresponding optimization variables.

For the optimization we employ the Broyden-Fletcher-Goldfarb-Shanno algorithm with box constraints on the variables (short L-BFGS-B) [11]. The solver minimizes the energy while respecting the positivity of the attenuations throughout the iterative optimization. L-BFGS-B implements a quasi Newton method, therefore, we need to provide the derivatives of the energy with respect to the unknown intensities *I*, the attenuation coefficients *α*, and the bleaching factor *β*_2_. These are given by

(7) $$\begin{array}{lcr} \frac{\mathrm{\delta E}\left(\alpha ,I,\beta_{2}\right)}{\mathrm{\delta I}\left(x\right)} & = & -2\overset{2}{\underset{i=1}{\sum }}\frac{\beta_{i}D_{i}\left(x\right)C_{i}^{2}\left(x\right)}{m^{2}I_{i}\left(x\right)+\sigma^{2}} \end{array}$$

(8) $$\begin{array}{lc} \frac{\mathrm{\delta E}\left(\alpha ,I,\beta_{2}\right)}{\delta \alpha \left(x\right)} & =\;4\sum_{i=1}^{2}\beta_{i}\int_{S}s_{i}\left(r\right)\int_{0}^{\infty }\frac{I\left(x-\ell r\right)T_{r}\left(x-\ell r\right)C_{i}\left(x-\ell r\right)D_{i}\left(x-\ell r\right)}{m^{2}I_{i}\left(x-\ell r\right)+\sigma^{2}}\;d\ell \;dr\\ & \;-\lambda \cdot 2\cdot \text{div}\left(\psi^{\prime }\left({∥\nabla \alpha \left(x\right)∥}^{2}\right)\nabla \alpha \left(x\right)\right)+\mu \frac{\alpha \left(x\right)}{\sqrt{\alpha^{2}\left(x\right)+\varepsilon_{\text{sp}}^{2}}} \end{array}$$

(9) $$\begin{array}{lcr} \frac{\mathrm{\partial E}\left(\alpha ,I,\beta_{2}\right)}{\partial \beta_{2}} & = & -2\underset{\Omega }{\int }\frac{D_{2}\left(x\right)I\left(x\right)C_{2}^{2}\left(x\right)}{m^{2}I_{2}\left(x\right)+\sigma^{2}}\;dx\;, \end{array}$$

where the derivative of the loss function *ψ*TM′(*x*^2^)=1 for the TM regularization and $\psi_{\text{TV}}^{\prime }\left(x^{2}\right)=\frac{1}{2\sqrt{x^{2}+\varepsilon_{\text{TV}}^{2}}}$ for the TV approximation. The detailed derivations are given in the Additional file 1.

The bleaching factor *β*_2_ prevents a direct analytic optimization of the intensities at each quasi Newton iteration as presented in [7]. Instead we optimize the bleaching factor *β*_2_ and intensities *I* within each quasi Newton iteration in an inner fixed-point iteration loop that alternates between analytic computation of *I*^[*j*]^ given $\beta_{2}^{\left[j-1\right]}$ and $\beta_{2}^{\left[j\right]}$ given *I*^[*j*]^ in inner iteration *j* where

(10) $$\begin{array}{cc} \;I^{\left[j\right]}\left(x\right) & =\frac{I_{1}\left(x\right)C_{1}^{2}\left(x\right)\left(m^{2}I_{2}\left(x\right)\;+\;\sigma^{2}\right)\;+\;\beta_{2}^{\left[j-1\right]}I_{2}\left(x\right)C_{2}^{2}\left(x\right)\left(m^{2}I_{1}\left(x\right)+\sigma^{2}\right)}{C_{1}^{4}\left(x\right)\left(m^{2}I_{2}\left(x\right)+\sigma^{2}\right)+{\beta_{2}^{\left[j-1\right]}}^{2}C_{2}^{4}\left(x\right)\left(m^{2}I_{1}\left(x\right)+\sigma^{2}\right)} \end{array}$$

(11) $$\begin{array}{cc} \beta_{2}^{\left[j\right]} & =\frac{\underset{\Omega }{\int }\frac{I_{2}\left(x\right)I^{\left[j\right]}\left(x\right)C_{2}^{2}\left(x\right)}{m^{2}I_{2}\left(x\right)+\sigma^{2}}\;dx}{\underset{\Omega }{\int }\frac{{\left(I^{\left[j\right]}\left(x\right)C_{2}^{2}\left(x\right)\right)}^{2}}{m^{2}I_{2}\left(x\right)+\sigma^{2}}\;dx}\;. \end{array}$$

### Implementation

The variational attenuation correction was implemented in C++ and run under Linux (Ubuntu 12.04) on an Intel Xeon E5-2680 (2.7GHz) Dual-Processor system. For the optimization we used the ready FORTRAN implementation of the L-BFGS-B optimizer. One iteration for data sub-sampled to 80×80×80 voxels needed on average 1.8 seconds, so a full reconstruction can be computed in the range of a few minutes. The complexity scales linearly with the number of voxels to process within each iteration (Additional file 1: Figure S3). The memory complexity also scales linearly with the raw data volume. Both quantities can be limited by sub-sampling the high resolution raw data. This has two advantages: First, less computational resources are needed and second, the weighted averaging during the sub-sampling already considerably reduces the image noise. The cone transmission is computed in parallel for all ray directions leading to a significant speed-up of the confocal microscope simulation. Depending on the random computation order introduced by the scheduling the results can slightly deviate from the numbers reported in the Results section. For real-world data we observed deviations of the estimated intensities of up to 3% after convergence of the algorithm. However, these differences are visually not recognizable.

#### Discrete derivative and integral computation

The gradients needed in the derivatives of the TM smoothness term and in the sparsity term are computed using central differences. For TV regularization we extended the numerical differentiation-scheme from [12] to 3-D to obtain the divergence term in the derivative of the smoothness term. The corresponding equations are given in the Additional file 1. The cone integrals are approximated as in [7] with a ray spacing of six degrees. This approximation of the cone integrals requires high regularization to lead to good reconstructions. We also did experiments with an alternative ray integration scheme that uses thin rays instead of the incrementally widening conic rays of [7]. To still capture all attenuations the ray sampling was increased so that the cone is sampled densely at the largest cone diameter with respect to the volume grid. The result is given in the Additional file 1 and shows, that the energy formulation leads to the desired solution but the numerical approximations have crucial influence on the resulting reconstruction.

### Data generation

#### Synthetic data

**Figure 3:**
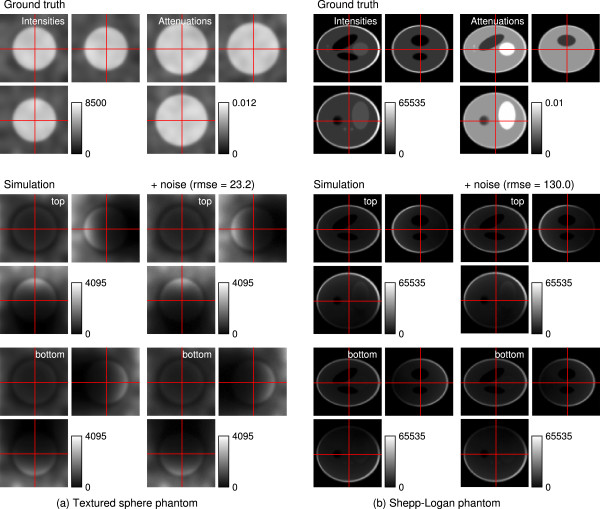
**Simulated data.** Synthetic ground truth and top/bottom simulations. **(a)** solid sphere with smooth random texture added to attenuations and intensities (well-posed reconstruction problem). Intensity range: [0, 8500], attenuation range: [0, 0.012]; **(b)** Shepp-Logan phantom with large constant areas (ill-posed reconstruction problem). Intensity range: [0, 65535], attenuation range: [0, 0.01]. All views show the central xy-, xz-, and zy-sections of the corresponding 3D volumes. Each panel shows: First row: ground truth intensities *I* (left) and absorption coefficients *α* (right). Second/third row: confocal simulations from top/bottom; Noise free (left); with applied Poisson-Gaussian noise (right) (*m*=0.05, *σ*=10. The rmse compared to the noise free simulation is indicated).

To quantitatively evaluate our method, we generated two different synthetic datasets. The first consists of a solid sphere with constant absorption coefficients of 0.006 per voxel. The interior 60% of the sphere were set to an intensity value of 4094. We added a smooth random texture with a variance of approximately 30% of the maximum of the corresponding quantity. This corresponds to the “well-posed” case when intensity and absorption information is available in the whole domain. The second dataset is the well-known Shepp-Logan phantom [13] consisting of a set of overlapping ellipsoids with homogeneous intensities. We assigned absorption coefficients to the different regions avoiding direct correlation with the intensities. Some regions were assigned equal attenuations independent of their intensity difference. During the simulation we applied an anisotropic Gaussian smoothing to reflect the microscope’s point spread function and ensure Nyquist sampling. For both datasets two recordings from opposite sides were simulated using (1) modeling absorption and photo bleaching (*β*_2_=0.8, meaning 20% signal loss between the recordings). Then Poisson noise with scaling *m*=0.05 and Gaussian noise with standard deviation *σ*=10 were applied. The datasets are shown in Figure 3.

#### Zebrafish

To show that the approach also copes well with real world data, we tested it on samples of the ViBE-Z database consisting of confocal recordings of whole zebrafish (Danio rerio) embryos, which were fixed 72h after fertilization. Sample preparation, recording setup and image preprocessing are described in detail in [7]. The processing was performed on sub-sampled data with isotropic voxel extents of 8 *μ*m.

#### *Arabidopsis thaliana*

Finally we tested the approach on recordings of the root tip of the model plant *Arabidopsis thaliana*. The samples were fixed 96h after germination and the cell membranes marked with an Alexa antibody stain. Then they were embedded in SlowFade Gold Antifade (Invitrogen) and recorded from top and bottom using a confocal microscope equipped with a 40 × oil immersion objective. We applied the elastic registration algorithm from [7] to register the two views to each other. Finally we performed a background subtraction prior to applying the attenuation correction. The embedding medium had a refractive index of 1.42 compared to a refractive index of the immersion oil of 1.52 for which the lens was adjusted. The attenuation correction was performed on sub-sampled data with isotropic voxel extents of 2 *μ*m.

### Parameter setup

We want all terms in the energy to have approximately the same influence on the optimization process. This leads to rough rules of thumb for the selection of *λ* and *μ*. Since all terms integrate over the whole image domain, the choice is independent of the number of voxels. The energy contribution of the data term is in the order of the squared expected intensity differences between recording and simulation divided by the Poisson weights. The smoothness term’s contribution is in the order of the magnitude of the expected attenuation gradient (TV) or its square (TM). Finally, the sparsity term’s contribution is in the order of the expected attenuations. E.g. for intensity data with an expected residual intensity difference of 5 (corresponding to the average noise intensity) and pure Gaussian noise with expected attenuation coefficients of 0.005 and gradient magnitudes of 0.0005 initial choices of $\lambda =\frac{5^{2}}{0.0005^{2}}=4\cdot 10^{8}$ and $\mu =\frac{5^{2}}{0.005}=5000$ (TM), resp. $\lambda =\frac{5^{2}}{0.0005}=5\cdot 10^{4}$ and *μ*=5000 (TV) are appropriate. The approximate estimates for the expected attenuations and their gradients were empirically confirmed on real world samples. For higher Poisson weighting *m* the factors have to be decreased accordingly. The optimal values depend on the image content and should be optimized for specific types of data.

For the synthetic phantoms we chose for each fixed *m* the optimal *λ* and *μ* which minimize the root mean squared error (rmse) of the true intensities and the estimated intensities. The parameters were empirically determined with an exponential grid search over a parameter range of *λ*∈{0,10^0^,…,10^9^} and *μ*∈{0,10^2^, 10^3^,10^4^} for the textured sphere phantom, and *λ*∈{0, 10^6^,…,10^12^} and *μ*∈{0,10^3^,…,10^8^} for the Shepp-Logan phantom. For all experiments we set *σ*:=1. For the real world data we used a conservative parameter set of *λ*=10^7^, *μ*=0 and *m*=0.1 (Arabidopsis) or *λ*=10^8^, *μ*=10^4^ and *m*=0 (zebrafish) for all experiments with TM regularization. For the zebrafish experiments with TV regularization we set *λ*=5·10^4^, *μ*=0, and *m*=0. For the real world data we stopped the iterative process when the visually optimal reconstruction of the intensities was reached, which was after between 3 to 20 iterations. For the textured sphere phantom data we set a maximum of 50 iterations for TM regularization and ran the algorithm to convergence for TV regularization. All results reported for the Shepp-Logan phantom were reached at convergence of the algorithm.

## Results and discussion

In Figure 2 the influence of the different extensions to the original model in [7] are summarized. If no prior knowledge about the attenuations is introduced (Figure 2 (c)) the approach is already able to reasonably reconstruct the original intensities. However, the attenuation field is coarse and cannot be applied to the reconstruction of secondary channels. With regularization (Figure 2 (d) and (e)) the attenuation field is much smoother, but especially with Tikhonov Miller regularization strong spurious attenuations outside the sample are estimated. Application of the sparsity term reduces these attenuation estimates (Figure 2 (f) and (g)). The residual apparent “bleeding” of the attenuation coefficients below the sample are the effect of different mean intensities in the top and bottom recordings, as e.g. introduced by photo bleaching. When this factor is additionally estimated during the optimization, the lower boundary becomes much clearer.

Detailed evaluation of the proposed model on the data sets described in the methods section and a comparison to [7] are given in the remainder of this section.

### Synthetic data

As a first baseline measure we computed the best possible outcome of the traditional one-factor-per-slice methods using the ground truth intensities of the synthetically generated phantoms. I.e. no method that assumes the correction factors to be a function of the z-position in the recorded volume can perform better than this. The optimal correction factor for each slice was computed by minimizing the rmse of the estimated intensities compared to the true intensities. The reconstruction error for slice *Ω*_*z*_⊂*Ω* is given by

(12) $$E_{z}\left(c_{1},c_{2}\right):=\overset{2}{\underset{i=1}{\sum }}\underset{x\in \Omega_{z}}{\sum }{\left(c_{i}\cdot I_{i}\left(x\right)-Î\left(x\right)\right)}^{2}\;,$$

where the *c*_*i*_∈*ℝ* are the correction factors for the top- and bottom-view and Î are the true intensities. The corresponding optimal correction factors *c*_1_ and *c*_2_ can be computed analytically to

(13) $$c_{i}=\frac{\underset{x\in \Omega_{z}}{\sum }Î\left(x\right)I_{i}\left(x\right)}{\underset{x\in \Omega_{z}}{\sum }I_{i}^{2}\left(x\right)}\;.$$

**Figure 4:**
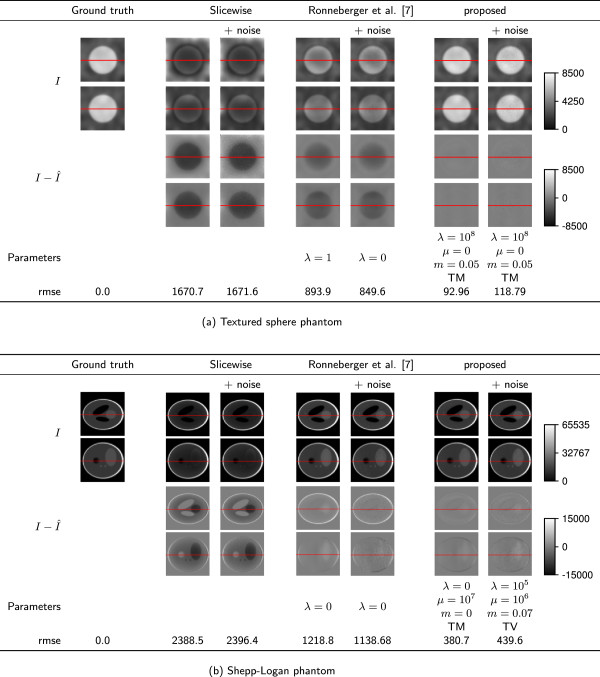
**Reconstruction results on simulated data.(a)** Textured sphere phantom; **(b)** Shepp-Logan phantom. Each vertically aligned pair of images shows an xy- and an xz-cut through the same volume. The cut position is indicated by the red line. Rows (top to bottom): *I*: real/estimated intensities, I−Î: difference of the reconstruction to the real intensities, **Parameters**: Used parameters, **rmse**: root mean squared error of the reconstructed intensities compared to the true intensities.

The reconstructions are shown in the column “Slicewise” of Figure 4. In both cases the one-factor-per-slice model was not able to reconstruct the interior intensities even though the true intensities were given.

As second baseline we applied the attenuation correction from [7] to all datasets and empirically determined the best regularization parameter *λ* for each of them. We also empirically optimized the parameters *λ* and *μ* for our proposed scheme using the exponential grid introduced in the parameter setup section. The best results regarding the rmse of the intensities are given in Tables 1 and 2 and are depicted in Figure 4.

**Table 1:** Results for the textured sphere phantom (+ noise)

**Method**	**m**	***λ***	***μ***	**E**	**nIter**	***β***	**rms**_***I***_	**rms **_***α***_
Ref. [7]	0	0	0	37.8917	19	1.0	849.58	0.0032
proposed (TM) (*β*_2_:=1)	0.05	10^6^	0	20.6261	50	1.0	319.80	0.0067
proposed (TM)	0	10^9^	10^2^	20.7742	50	0.80	177.79	0.0006
proposed (TM)	0.02	10^9^	10^4^	20.3511	50	0.80	154.72	0.0006
proposed (TM)	0.05	10^8^	10^3^	12.7709	50	0.80	**117.31**	**0.0004**
proposed (TM)	0.07	10^8^	10^3^	10.8522	50	0.80	127.17	**0.0004**
proposed (TM)	0.1	10^6^	10^3^	8.2228	50	0.80	130.29	0.0005
proposed (TV)	0	5·10^3^	0	13.4404	89	0.80	156.14	0.0033
proposed (TV)	0.02	10^4^	10^3^	13.0192	104	0.80	185.30	0.0028
proposed (TV)	0.05	5·10^4^	10^3^	12.188	68	0.80	116.77	0.0008
proposed (TV)	0.07	5·10^4^	10^3^	10.3538	79	0.80	**115.98**	**0.0007**
proposed (TV)	0.1	5·10^4^	10^2^	8.1756	127	0.80	119.06	**0.0007**

*Abbreviations*: G = Gauss, P = Poisson, TM = Tikhonov-Miller, TV = Total Variation, E = Final energy after convergence, nIter = Iterations till convergence.

**Table 2:** Results for the Shepp-Logan phantom (+ noise)

**Method**	**m**	***λ***	***μ***	**E**	**nIter**	***β***	**rms**_***I***_	**rms **_***α***_
Ref. [7]	0	0	0	84.2003	88	1.0	1138.68	0.0021
proposed (TM) (*β*_2_:=1)	0.05	10^10^	10^6^	72.1663	117	1.0	822.27	0.0011
proposed (TM)	0	10^6^	10^7^	152.712	770	0.80	639.52	0.0032
proposed (TM)	0.02	10^8^	10^7^	148.08	268	0.80	518.18	0.0019
proposed (TM)	0.05	10^9^	10^6^	68.2854	66	0.80	487.45	0.0010
proposed (TM)	0.07	10^9^	10^6^	63.4456	48	0.80	**449.03**	**0.0008**
proposed (TM)	0.1	10^8^	10^6^	57.677	65	0.80	490.76	0.0010
proposed (TV)	0	5·10^6^	10^7^	166.514	94	0.80	466.14	**0.0008**
proposed (TV)	0.02	5·10^5^	10^7^	151.261	75	0.80	470.12	0.0009
proposed (TV)	0.05	10^5^	10^6^	67.4981	88	0.80	567.72	0.0013
proposed (TV)	0.07	10^5^	10^6^	63.3558	38	0.80	**439.59**	**0.0008**
proposed (TV)	0.1	10^5^	10^6^	58.1152	40	0.80	478.66	**0.0008**

*Abbreviations*: G = Gauss, P = Poisson, TM = Tikhonov-Miller, TV = Total Variation, E = Final energy after convergence, nIter = Iterations till convergence.

For the synthetic data, the new model clearly outperforms the baseline from [7] even for sub-optimal choices of the Poisson weight *m*. The increase in performance is clearer for the textured phantom in which our approximation to the real noise is less affected by suboptimal mean value estimates in low-intensity regions, but even for the Shepp-Logan phantom the reconstruction quality is increased almost by a factor of three. The noise model and the bleaching factor *β*_2_ both affect the reconstruction significantly. The sparsity term also plays an important role for the reconstruction in two ways: Firstly, it avoids high attenuation estimates in regions with insufficient information; Secondly, it suppresses errors introduced by the discrete numerical approximations.

**Figure 5:**
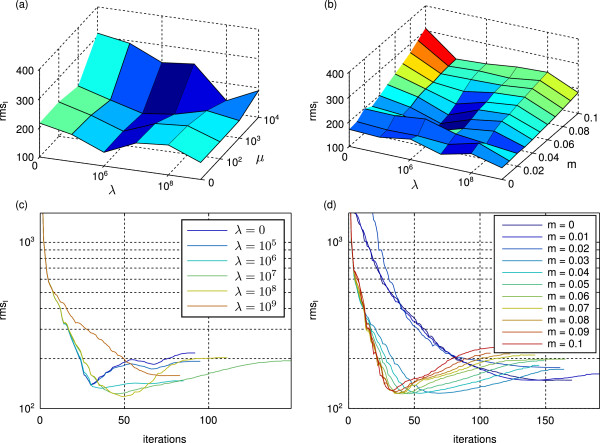
**Effects of different choices for*λ*,*μ*, and*m* on the rmse of the reconstructed intensities of the textured sphere phantom using TM regularization.(a)** Effect of different combinations of *λ* and *μ* on the reconstruction. Residual parameters: *m*=0.05, nIter = 50. **(b)** Effect of different combinations of *λ* and *m* on the reconstruction. Residual parameters: *μ*=0, nIter = 50. **(c)** Evolution of the rmse of the intensities during the iterative process for different choices of *λ*. Residual parameters: *μ*=0, *m*=0.05. **(d)** Evolution of the rmse of the intensities during the iterative process for different choices of *m*. Residual parameters: *λ*=10^7^, *μ*=0.

**Figure 6:**
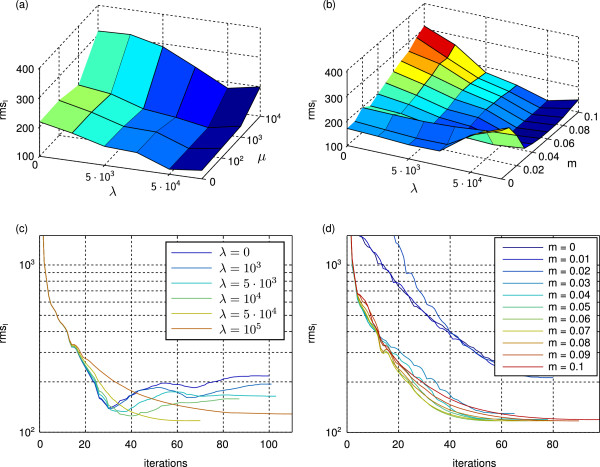
**Effects of different choices for*λ*,*μ*, and*m* on the rmse of the reconstructed intensities of the textured sphere phantom using TV regularization.(a)** Effect of different combinations of *λ* and *μ* on the reconstruction. Residual parameters: *m*=0.05, run to convergence. **(b)** Effect of different combinations of *λ* and *m* on the reconstruction. Residual parameters: *μ*=0, run to convergence. **(c)** Evolution of the rmse of the intensities during the iterative process for different choices of *λ*. Residual parameters: *μ*=0, *m*=0.05. **(d)** Evolution of the rmse of the intensities during the iterative process for different choices of *m*. Residual parameters: *λ*=5·10^4^, *μ*=0.

We evaluated the reconstruction quality with respect to the three parameters *λ*, *μ*, and *m* (TV: Figure 5 (a) and (b), TM: Figure 6 (a) and (b)). As quality measure we used the rmse of the estimated intensities. We found that the results are stable over a wide range of parameters. The parameter having the highest impact on the result is the smoothness weight *λ*, followed by *m* and finally *μ*. We also evaluated the evolution of the rmse during the optimization process for different choices of the parameters (TM: Figure 5 (c) and (d), TV: Figure 6 (c) and (d)). For all parameter choices the rmse first decreases rapidly reaching a very good reconstruction after 30 to 60 iterations (From practical observations we found that for real world data the optimum is reached earlier). Beyond that point the boundary effects and inaccuracies in the applied numerical approximations lead to an increase in the rmse for the TM regularization. For high TV regularization the attenuations are well localized within the sample volume and therefore no significant attenuations are estimated at the boundaries. This results in monotonically decreasing rmses with small local fluctuations.

### Zebrafish

**Figure 7:**
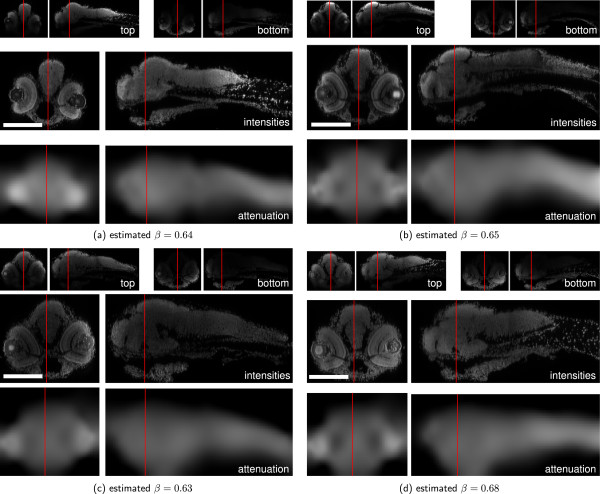
**Attenuation Correction on zebrafish data using TM regularization.** Result of the application of the proposed method to four challenging samples of the ViBE-Z database (TM regularized, *λ*=10^8^, *μ*=10^4^, *m*=0). **(a-d)**: Reconstruction results for four different zebarfish larvae. Top: yz- and xz-cuts through raw recordings from top and bottom, middle: Reconstructed intensities, bottom: Estimated attenuation coefficients. Red lines indicate the cut positions of the corresponding views. Scale bars: 200 *μ*m.

**Figure 8:**
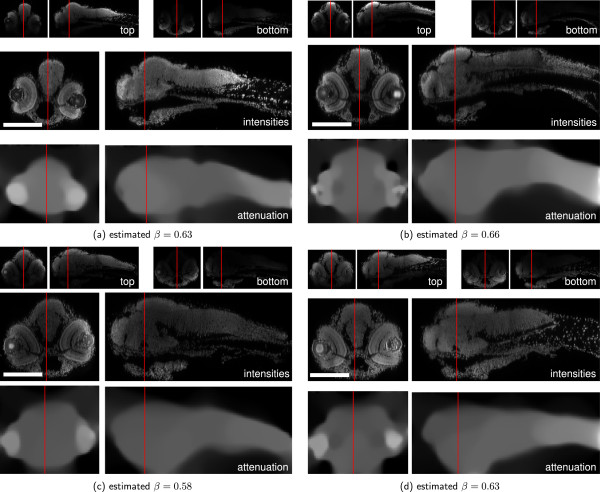
**Attenuation Correction on zebrafish data using TV regularization.** Result of the application of the proposed method to four challenging samples of the ViBE-Z database (TV regularized, *λ*=5·10^4^, *μ*=0, *m*=0). **(a-d)**: Reconstruction results for four different zebarfish larvae. Top: yz- and xz-cuts through raw recordings from top and bottom, middle: Reconstructed intensities, bottom: Estimated attenuation coefficients. Red lines indicate the cut positions of the corresponding views. Scale bars: 200 *μ*m.

Additionally to the result shown in Figure 1, we applied our method to other zebrafish samples with varying staining quality. The reconstructions with Tikhonov Miller regularization are shown in Figure 7 and for total variation regularization in Figure 8. The estimated attenuation coefficients clearly resemble the shapes of the embryos. The bright spots in the eyes stem from the strong refraction of the eyes’ lenses showing the modeling limitations of the presented approach. However, due to the imposed priors the artifact is localized in a small region and affects the surrounding reconstruction only marginally.

**Figure 9:**
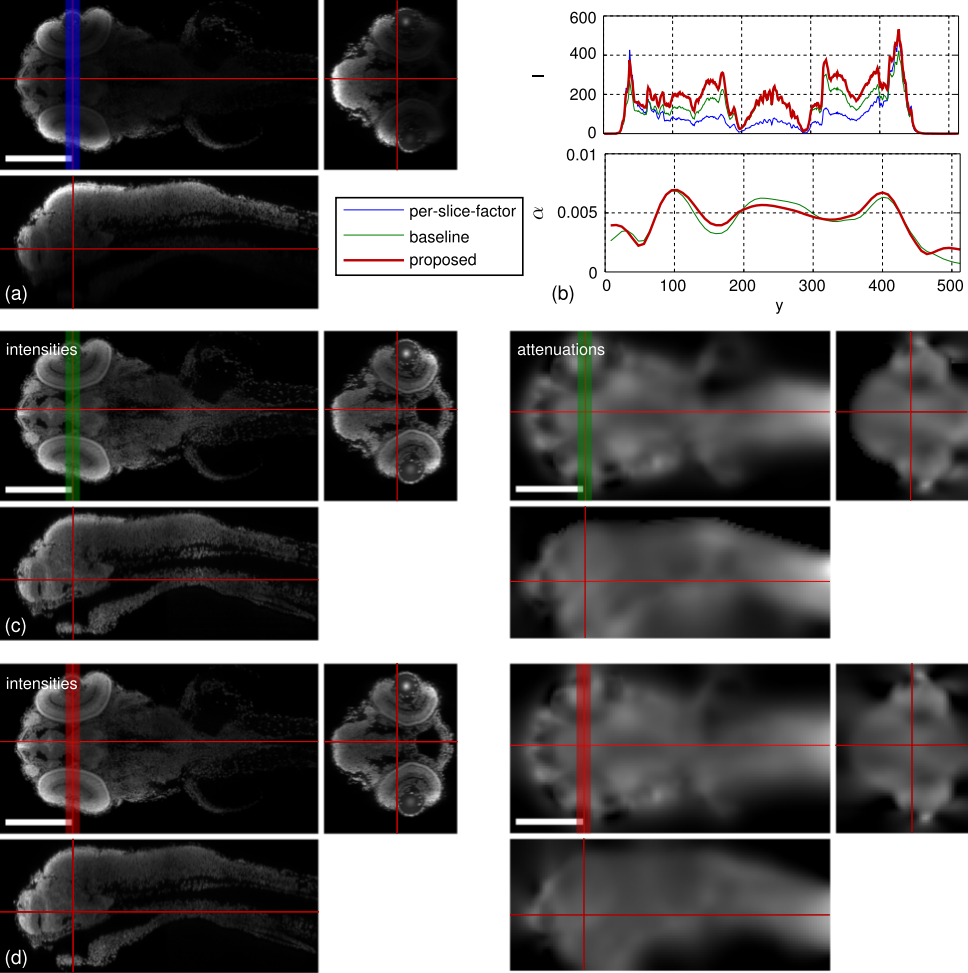
**Comparison of the proposed method with **[7]** and the one-factor-per-slice model on one zebrafish dataset.** Each panel shows xy-, xz- and zy-cuts through the volume. The cut positions are indicated by the red lines. **(a)** Raw recording with scaled intensities to match the intensities of the reconstruction on the eye surface. **(b)** Averaged intensity and attenuation profiles along the y-direction of the xy cuts. Cut positions and averaging width are indicated by colored bars. **(c)** Baseline reconstruction [7]. **(d)** Proposed method. Scale bars: 200 *μ*m.

Figure 9 shows a comparison of the proposed approach to the one-factor-per-slice model and the baseline approach [7] for one fish. For [7] we set *λ*=10^7^, for the proposed approach we used *λ*=10^7^, *μ*=10^3^ and *m*=0. The one-factor-per-slice model is not able to recover the intensity spectrum since it cannot change the ratio between the boundary and interior intensities. In the brain region of the fish the baseline and the proposed approach estimate the same intensities, whereas the proposed approach emphasizes the tissue layers in the zebrafish eyes stronger. The proposed approach shows slightly smaller intensity overshoots at the eyes’ surfaces and around the nose but overall both reconstructions are convincing. The apparent “bleeding” of the attenuation coefficients ventral to the fish is reduced. The staircasing artifacts along the back of the fish in the baseline approach which were introduced with the orthogonal subspace projections are effectively removed.

### *Arabidopsis thaliana*

**Figure 10:**
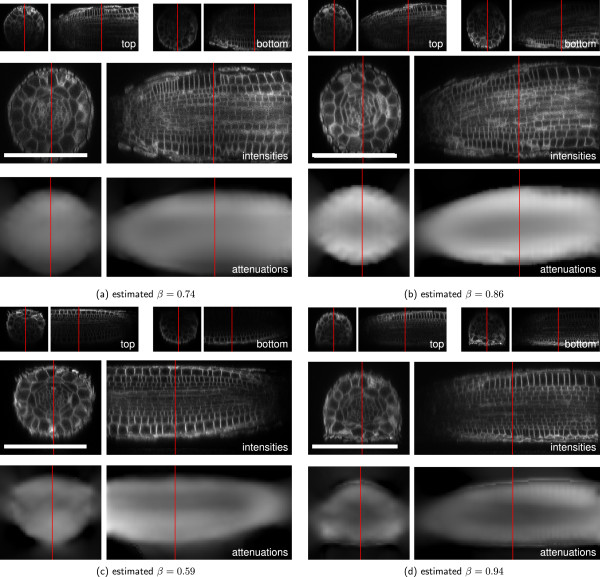
**Attenuation Correction on*Arabidopsis thaliana* data.** Result of the application of the proposed method to four Arabidopsis root tip samples (TM regularized, *λ*=10^7^, *μ*=0, *m*=0.1). **(a-d)**: Reconstruction results for four different Arabidopsis root tips. Top: yz- and xz-cuts through raw recordings from top and bottom, middle: Reconstructed intensities, bottom: Estimated attenuation coefficients. Red lines indicates the cut position of the corresponding view. Scale bars: 100 *μ*m.

The mismatch in refractive indices of immersion and embedding media in the Arabidopsis sample preparation leads to an aberration induced signal loss, that is not modeled in the presented approach. However, Figure 10 shows that our method still accurately reconstructs the intensities of the root tip datasets.

**Figure 11:**
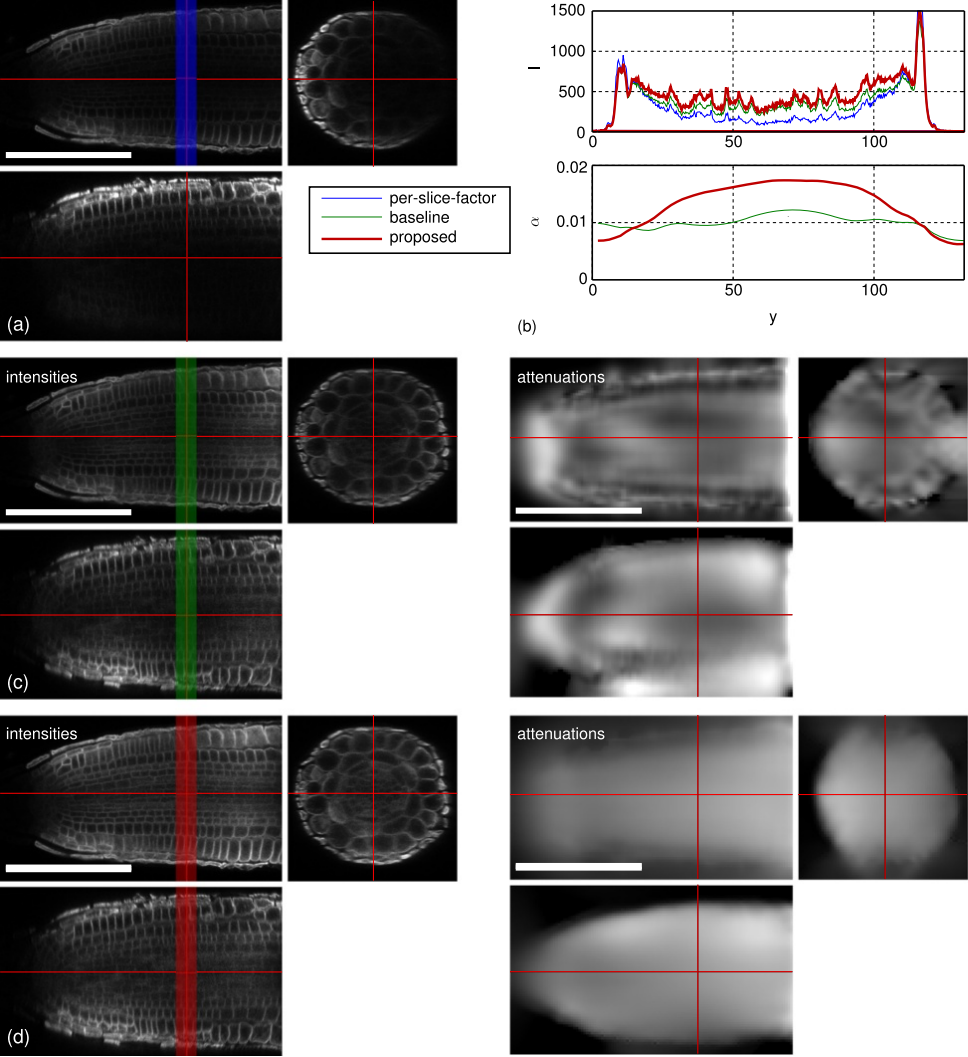
**Comparison of the proposed method with **[7]** and the one-factor-per-slice model on one Arabidopsis root tip dataset.** Each panel shows xy-, xz- and zy-cuts through the volume. The cut positions are indicated by the red lines. **(a)** Raw recording with scaled intensities to match the intensities of the reconstructions at the root boundary. **(b)** Averaged intensity profiles along the y-direction of the xy cuts. Cut positions and averaging widths are indicated by colored bars. **(c)** Baseline reconstruction [7]. **(d)** Proposed method. Scale bars: 100 *μ*m.

Figure 11 shows a comparison of the proposed approach to the one-factor-per-slice model and the baseline approach [7] for one root tip. Again the one-factor-per slice model cannot reconstruct the interior intensities. The reconstruction of [7] and the proposed approach both significantly enhance the root-internal contrast. However, an intensity gradient towards the root center remains visible. We assume, that it is induced by imperfect marker distributions due to incomplete tissue penetration and the properties of the inner cell membranes. The estimated attenuation field closely resembles the root’s shape and is homogeneous compared to the baseline approach. The baseline approach shows strong variation within the root and additionally estimates strong attenuation outside the root volume to cope with the bleaching effects. Such erroneous attenuation estimates may lead to reasonable reconstructions of the intensities of the channel they were estimated on, but they will fail in reconstructing secondary channels containing the markers to quantify like protein patterns.

### Limitations of the approach

The exponential decay model along a ray is only strictly valid for pure absorption. In most cases local random refractions can be also described by this model. However, in areas with clearly structured refraction, as e.g. in the eyes of the zebrafish, where the light is actively bundled, the model is violated and localized errors in the attenuation estimates are introduced. We minimize the influence of these errors with high regularization, however, a better modeling of refraction would be a desirable – though practically very challenging – extension.

Another source of error is the limited recording volume. Samples exceeding this volume introduce the problem of sensibly guessing the outside attenuations the rays pass before entering the recording volume. Boundary effects can lead to solutions with low energies which are qualitatively far away from the optimum, especially when performing many iterations. In our image formation model we assume zero outside attenuations (natural boundary conditions), while for the regularization we assume Neumann boundary conditions. If possible, the recording volume should be increased to contain more background in cases of boundary problems. If this is not possible the TV regularization with its sharp boundaries is to prefer over the TM regularization. Additionally a high weight on the sparsity term alleviates effects that lead to extreme attenuation estimates. This can be the case when outside attenuations are explained by a thin highly absorbing region at the image boundary. An alternative, that leads to visually good, but energetically suboptimal results, is to restrict the number of iterations (less than ten iterations usually lead to qualitatively good results). This has the additional advantage of very low computation times.

## Conclusions

We could significantly improve the results of the variational attenuation correction presented in [7] by additionally modeling photo bleaching and replacing the ad-hoc Gaussian noise assumption by the (approximate) Poisson-Gaussian statistics. The choice of the loss function in the smoothness term allows to choose between smoothly varying (TM) or piecewise constant (TV) attenuation fields. The choice of the appropriate regularization is application dependent. In our case both regularization strategies lead to equally plausible results in the rather inhomogeneous biological samples analyzed. TV regularization is more stable in practice because the attenuation is much better localized, and therefore less boundary artifacts – that may lead to convergence to undesired solutions – are introduced. For both regularizations the sparsity term also actively avoids boundary errors, by keeping the attenuation field compact. However, high sparsity weights lead to an underestimation of the attenuation volume and should be avoided. Instead, an earlier manual termination of the iterative process leads to very good results without introducing this side-effect.

We showed the efficacy of the presented method on highly complex real world examples, where it was able to significantly increase the homogeneity of the measured signal and attenuation fields. This is crucial if the attenuation field is used to correct secondary channels containing sparse structures within the anatomy. Based on these findings, we conclude that the presented attenuation correction approach is an important step towards the quantification of confocal microscopic data.

## Competing interests

The authors declare that they have no competing interests.

## Authors’ contributions

TS, MK and OR developed the theory. TS and OR performed the implementation and did the experiments. JD and TB prepared the Arabidopsis samples and did the recordings. KP initiated the plant project. TS, MK and OR wrote the paper. All authors read and approved the final manuscript.

## Supplementary Material

Additional file 1 Supplementary material. Click here for file
