# Supplementary material for: Variational attenuation correction in two-view confocal microscopy
Source: BMC Bioinformatics. 2013 Dec 18;14:366. doi: 10.1186/1471-2105-14-366 (PMC3878336; doi:10.1186/1471-2105-14-366)
Supplement: Additional file 1 — Supplementary material. [file 1471-2105-14-366-S1.pdf]

Supplementary Material to "Variational Attenuation Correction in Two-View Confocal Microscopy"

Computation of the Energy Derivatives

The first variations of the intermediate formulas wrt. the estimated intensities I are

$$
\begin{aligned}
\frac{\mathrm{d}}{\mathrm{d}\epsilon} F_i\left[\alpha, I+\epsilon h, \beta_2\right](\mathbf{x})\Big|_{\epsilon=0} &= \frac{\mathrm{d}}{\mathrm{d}\epsilon} \beta_i\left(I(\mathbf{x})+\epsilon h(\mathbf{x})\right) C_i^2[\alpha](\mathbf{x})\Big|_{\epsilon=0} = \beta_i C_i^2[\alpha](\mathbf{x}) \cdot h(\mathbf{x}) \\
\frac{\mathrm{d}}{\mathrm{d}\epsilon} D_i\left[\alpha, I+\epsilon h, \beta_2\right](\mathbf{x})\Big|_{\epsilon=0} &= \frac{\mathrm{d}}{\mathrm{d}\epsilon}\left(I_i(\mathbf{x})-F_i\left[\alpha, I+\epsilon h, \beta_2\right](\mathbf{x})\right)\Big|_{\epsilon=0} = -\frac{\mathrm{d}}{\mathrm{d}\epsilon} F_i\left[\alpha, I+\epsilon h, \beta_2\right](\mathbf{x})\Big|_{\epsilon=0} \\
&= -\beta_i C_i^2[\alpha](\mathbf{x}) \cdot h(\mathbf{x})
\end{aligned}
$$

and wrt. the estimated attenuation coefficients α

$$
\begin{aligned}
\frac{\mathrm{d}}{\mathrm{d}\epsilon} T_{\mathbf{r}}[\alpha+\epsilon h](\mathbf{x})\Big|_{\epsilon=0} &= \frac{\mathrm{d}}{\mathrm{d}\epsilon} e^{-\int_0^\infty \alpha(\mathbf{x}+\ell\mathbf{r})+\epsilon h(\mathbf{x}+\ell\mathbf{r})\,\mathrm{d}\ell}\Big|_{\epsilon=0} \\
&= -e^{-\int_0^\infty \alpha(\mathbf{x}+\ell\mathbf{r})+\epsilon h(\mathbf{x}+\ell\mathbf{r})\,\mathrm{d}\ell} \frac{\mathrm{d}}{\mathrm{d}\epsilon} \int_0^\infty \alpha(\mathbf{x}+\ell\mathbf{r})+\epsilon h(\mathbf{x}+\ell\mathbf{r})\,\mathrm{d}\ell\Big|_{\epsilon=0} \\
&= -T_{\mathbf{r}}[\alpha](\mathbf{x}) \int_0^\infty h(\mathbf{x}+\ell\mathbf{r})\,\mathrm{d}\ell \\
\frac{\mathrm{d}}{\mathrm{d}\epsilon} C_i[\alpha+\epsilon h](\mathbf{x})\Big|_{\epsilon=0} &= \frac{\mathrm{d}}{\mathrm{d}\epsilon} \int_S s_i(\mathbf{r}) \cdot T_{\mathbf{r}}[\alpha+\epsilon h](\mathbf{x})\,\mathrm{d}\mathbf{r}\Big|_{\epsilon=0} = \int_S s_i(\mathbf{r}) \cdot \frac{\mathrm{d}}{\mathrm{d}\epsilon} T_{\mathbf{r}}[\alpha+\epsilon h](\mathbf{x})\,\mathrm{d}\mathbf{r}\Big|_{\epsilon=0} \\
&= -\int_S s_i(\mathbf{r})\, T_{\mathbf{r}}[\alpha](\mathbf{x}) \int_0^\infty h(\mathbf{x}+\ell\mathbf{r})\,\mathrm{d}\ell\,\mathrm{d}\mathbf{r} \\
\frac{\mathrm{d}}{\mathrm{d}\epsilon} F_i\left[\alpha+\epsilon h, I, \beta_2\right](\mathbf{x})\Big|_{\epsilon=0} &= \beta_i I(\mathbf{x}) \frac{\mathrm{d}}{\mathrm{d}\epsilon} C_i^2[\alpha+\epsilon h](\mathbf{x})\Big|_{\epsilon=0} = 2\beta_i I(\mathbf{x})\, C_i[\alpha+\epsilon h](\mathbf{x}) \frac{\mathrm{d}}{\mathrm{d}\epsilon} C_i[\alpha+\epsilon h](\mathbf{x})\Big|_{\epsilon=0} \\
&= -2\beta_i I(\mathbf{x})\, C_i[\alpha](\mathbf{x}) \int_S s_i(\mathbf{r})\, T_{\mathbf{r}}[\alpha](\mathbf{x}) \int_0^\infty h(\mathbf{x}+\ell\mathbf{r})\,\mathrm{d}\ell\,\mathrm{d}\mathbf{r} \\
\frac{\mathrm{d}}{\mathrm{d}\epsilon} D_i\left[\alpha+\epsilon h, I, \beta_2\right](\mathbf{x})\Big|_{\epsilon=0} &= \frac{\mathrm{d}}{\mathrm{d}\epsilon}\left(I_i(\mathbf{x})-F_i\left[\alpha+\epsilon h, I, \beta_2\right](\mathbf{x})\right)\Big|_{\epsilon=0} = -\frac{\mathrm{d}}{\mathrm{d}\epsilon} F_i\left[\alpha+\epsilon h, I, \beta_2\right](\mathbf{x})\Big|_{\epsilon=0} \\
&= 2\beta_i I(\mathbf{x})\, C_i[\alpha](\mathbf{x}) \int_S s_i(\mathbf{r})\, T_{\mathbf{r}}[\alpha](\mathbf{x}) \int_0^\infty h(\mathbf{x}+\ell\mathbf{r})\,\mathrm{d}\ell\,\mathrm{d}\mathbf{r} \\
&= 2\beta_i \int_S \int_0^\infty I(\mathbf{x})\, C_i[\alpha](\mathbf{x})\, s_i(\mathbf{r})\, T_{\mathbf{r}}[\alpha](\mathbf{x})\, h(\mathbf{x}+\ell\mathbf{r})\,\mathrm{d}\ell\,\mathrm{d}\mathbf{r}\,.
\end{aligned}
$$

The Gâteaux derivative of the data term becomes

$$
\begin{aligned}
\frac{\mathrm{d}}{\mathrm{d}\epsilon} E_{\text{data}}\left(\alpha+\epsilon h, I, \beta_2\right)\Big|_{\epsilon=0} &= \frac{\mathrm{d}}{\mathrm{d}\epsilon} \sum_{i=1}^{2} \int_\Omega \frac{D_i^2\left[\alpha+\epsilon h, I, \beta_2\right](\mathbf{x})}{m^2 I_i(\mathbf{x})+\sigma^2}\,\mathrm{d}\mathbf{x}\Big|_{\epsilon=0} \\
&= 4 \sum_{i=1}^{2} \beta_i \int_\Omega \frac{D_i\left[\alpha, I, \beta_2\right](\mathbf{x})}{m^2 I_i(\mathbf{x})+\sigma^2} \\
&\qquad \cdot \int_S \int_0^\infty I(\mathbf{x})\, C_i[\alpha](\mathbf{x})\, s_i(\mathbf{r})\, T_{\mathbf{r}}[\alpha](\mathbf{x})\, h(\mathbf{x}+\ell\mathbf{r})\,\mathrm{d}\ell\,\mathrm{d}\mathbf{r}\,\mathrm{d}\mathbf{x}
\end{aligned}
$$

We substitute $\mathbf{y} := \mathbf{x} + \ell\mathbf{r}$ and obtain

$$\begin{aligned}\left.\frac{\mathrm{d}}{\mathrm{d}\epsilon} E_{\text{data}}\left(\alpha + \epsilon h, I, \beta_2\right)\right|_{\epsilon=0} &= 4 \sum_{i=1}^{2} \beta_i \int_\Omega \int_S s_i\left(\mathbf{r}\right) \int_0^\infty \frac{D_i\left[\alpha, I, \beta_2\right]\left(\mathbf{y} - \ell\mathbf{r}\right)}{m^2 I_i\left(\mathbf{y} - \ell\mathbf{r}\right) + \sigma^2} \\ &\quad \cdot I\left(\mathbf{y} - \ell\mathbf{r}\right) C_i\left[\alpha\right]\left(\mathbf{y} - \ell\mathbf{r}\right) T_{\mathbf{r}}\left[\alpha\right]\left(\mathbf{y} - \ell\mathbf{r}\right) h\left(\mathbf{y}\right)\, \mathrm{d}\ell\, \mathrm{d}\mathbf{r}\, \mathrm{d}\mathbf{y}\,.\end{aligned}$$

The Gâteaux derivative of the smoothness term is given by

$$\begin{aligned}\left.\frac{\mathrm{d}}{\mathrm{d}\epsilon} E_{\text{smooth}}\left(\alpha + \epsilon h\right)\right|_{\epsilon=0} &= \left.\frac{\mathrm{d}}{\mathrm{d}\epsilon} \int_\Omega \psi\left(\left\|\nabla\left(\alpha + \epsilon h\right)\left(\mathbf{x}\right)\right\|^2\right) \mathrm{d}\mathbf{x}\right|_{\epsilon=0} \\ &= \left.\int_\Omega \psi'\left(\left\|\nabla\left(\alpha + \epsilon h\right)\left(\mathbf{x}\right)\right\|^2\right) \frac{\mathrm{d}}{\mathrm{d}\epsilon} \left\|\nabla\left(\alpha + \epsilon h\right)\left(\mathbf{x}\right)\right\|^2 \mathrm{d}\mathbf{x}\right|_{\epsilon=0} \\ &= \left.\int_\Omega \psi'\left(\left\|\nabla\left(\alpha + \epsilon h\right)\left(\mathbf{x}\right)\right\|^2\right) \frac{\mathrm{d}}{\mathrm{d}\epsilon} \sum_{d=1}^{3} \left(\frac{\partial}{\partial x_d}\left(\alpha + \epsilon h\right)\left(\mathbf{x}\right)\right)^2 \mathrm{d}\mathbf{x}\right|_{\epsilon=0} \\ &= \left. 2 \int_\Omega \psi'\left(\left\|\nabla\left(\alpha + \epsilon h\right)\left(\mathbf{x}\right)\right\|^2\right) \sum_{d=1}^{3} \frac{\partial}{\partial x_d}\left(\alpha + \epsilon h\right)\left(\mathbf{x}\right) \frac{\partial}{\partial x_d} \frac{\mathrm{d}}{\mathrm{d}\epsilon}\left(\alpha + \epsilon h\right)\left(\mathbf{x}\right) \mathrm{d}\mathbf{x}\right|_{\epsilon=0} \\ &= \left. 2 \int_\Omega \psi'\left(\left\|\nabla\left(\alpha + \epsilon h\right)\left(\mathbf{x}\right)\right\|^2\right) \sum_{d=1}^{3} \frac{\partial}{\partial x_d}\left(\alpha + \epsilon h\right)\left(\mathbf{x}\right) \frac{\partial}{\partial x_d} h\left(\mathbf{x}\right) \mathrm{d}\mathbf{x}\right|_{\epsilon=0} \\ &= 2 \int_\Omega \psi'\left(\left\|\nabla\alpha\left(\mathbf{x}\right)\right\|^2\right) \left(\nabla\alpha\left(\mathbf{x}\right)\right)^\top \nabla h\left(\mathbf{x}\right) \mathrm{d}\mathbf{x}\,.\end{aligned}$$

Using integration by parts, we obtain

$$= \quad -2 \int_\Omega \operatorname{div}\left(\psi'\left(\left\|\nabla\alpha\left(\mathbf{x}\right)\right\|^2\right) \nabla\alpha\left(\mathbf{x}\right)\right) h\left(\mathbf{x}\right) \mathrm{d}\mathbf{x} + 2\left[\left(\psi'\left(\left\|\nabla\alpha\left(\mathbf{x}\right)\right\|^2\right) \left(\mathbf{n}\left(\mathbf{x}\right)\right)^\top \nabla\alpha\left(\mathbf{x}\right)\right) h\left(\mathbf{x}\right)\right]_{\mathbf{x}\in\partial\Omega}\,.$$

where $\partial\Omega$ denotes the boundary and $\mathbf{n}$ the boundary normals.

For Tikhonov Miller ($\psi\left(s^2\right) = s^2$, $\psi'\left(s^2\right) = 1$) this simplifies to

$$-2 \int_\Omega \Delta\alpha\left(\mathbf{x}\right) h\left(\mathbf{x}\right) \mathrm{d}\mathbf{x} + 2\left[\left(\left(\mathbf{n}\left(\mathbf{x}\right)\right)^\top \nabla\alpha\left(\mathbf{x}\right)\right) h\left(\mathbf{x}\right)\right]_{\mathbf{x}\in\partial\Omega}\,,$$

and for the robust total variation approximation ($\psi\left(s^2\right) = \sqrt{s^2 + \epsilon_{\text{TV}}}$, $\psi'\left(s^2\right) = \frac{1}{2\sqrt{s^2 + \epsilon_{\text{TV}}^2}}$) we obtain

$$-\int_\Omega \operatorname{div}\left(\frac{\nabla\alpha\left(\mathbf{x}\right)}{\sqrt{\left\|\nabla\alpha\left(\mathbf{x}\right)\right\|^2 + \epsilon_{\text{TV}}^2}}\right) h\left(\mathbf{x}\right) \mathrm{d}\mathbf{x} + \left[\left(\left(\mathbf{n}\left(\mathbf{x}\right)\right)^\top \frac{\nabla\alpha\left(\mathbf{x}\right)}{\sqrt{\left\|\nabla\alpha\left(\mathbf{x}\right)\right\|^2 + \epsilon_{\text{TV}}^2}}\right) h\left(\mathbf{x}\right)\right]_{\mathbf{x}\in\partial\Omega}\,.$$

The Gâteaux derivative of the sparsity term is given by

$$\begin{aligned}
\frac{\mathrm{d}}{\mathrm{d}\epsilon} E_{\text{sparse}}\left(\alpha+\epsilon h\right)\Big|_{\epsilon=0} &= \frac{\mathrm{d}}{\mathrm{d}\epsilon}\int_{\Omega}\sqrt{\left(\alpha+\epsilon h\right)^{2}\left(\mathbf{x}\right)+\epsilon_{\text{sp}}^{2}}\,\mathrm{d}\mathbf{x}\Big|_{\epsilon=0} \\
&= \int_{\Omega}\frac{1}{2\sqrt{\left(\alpha+\epsilon h\right)^{2}\left(\mathbf{x}\right)+\epsilon_{\text{sp}}^{2}}}\frac{\mathrm{d}}{\mathrm{d}\epsilon}\left(\alpha+\epsilon h\right)^{2}\left(\mathbf{x}\right)\,\mathrm{d}\mathbf{x}\Big|_{\epsilon=0} \\
&= 2\int_{\Omega}\frac{1}{2\sqrt{\left(\alpha+\epsilon h\right)^{2}\left(\mathbf{x}\right)+\epsilon_{\text{sp}}^{2}}}\left(\alpha+\epsilon h\right)\left(\mathbf{x}\right)\frac{\mathrm{d}}{\mathrm{d}\epsilon}\left(\alpha+\epsilon h\right)\left(\mathbf{x}\right)\,\mathrm{d}\mathbf{x}\Big|_{\epsilon=0} \\
&= \int_{\Omega}\frac{\alpha\left(\mathbf{x}\right)}{\sqrt{\alpha^{2}\left(\mathbf{x}\right)+\epsilon_{\text{sp}}^{2}}}h\left(\mathbf{x}\right)\,\mathrm{d}\mathbf{x}\,.
\end{aligned}$$

Altogether this leads to the functional derivatives presented in the main paper

$$\begin{aligned}
\frac{\delta E\left(\alpha,I,\beta_{2}\right)}{\delta I\left(\mathbf{x}\right)} &= -2\sum_{i=1}^{2}\frac{\beta_{i}D_{i}\left(\mathbf{x}\right)C_{i}^{2}\left(\mathbf{x}\right)}{m^{2}I_{i}\left(\mathbf{x}\right)+\sigma^{2}} \\
\frac{\delta E\left(\alpha,I,\beta_{2}\right)}{\delta\alpha\left(\mathbf{x}\right)} &= 4\sum_{i=1}^{2}\beta_{i}\int_{S}s_{i}\left(\mathbf{r}\right)\int_{0}^{\infty}\frac{I\left(\mathbf{x}-\ell\mathbf{r}\right)T_{\mathbf{r}}\left(\mathbf{x}-\ell\mathbf{r}\right)C_{i}\left(\mathbf{x}-\ell\mathbf{r}\right)D_{i}\left(\mathbf{x}-\ell\mathbf{r}\right)}{m^{2}I_{i}\left(\mathbf{x}-\ell\mathbf{r}\right)+\sigma^{2}}\,\mathrm{d}\ell\,\mathrm{d}\mathbf{r} \\
&\quad -\lambda\cdot2\cdot\operatorname{div}\left(\psi'\left(\left\|\nabla\alpha\left(\mathbf{x}\right)\right\|^{2}\right)\nabla\alpha\left(\mathbf{x}\right)\right)+\mu\frac{\alpha\left(\mathbf{x}\right)}{\sqrt{\alpha^{2}\left(\mathbf{x}\right)+\epsilon_{\text{sp}}^{2}}} \\
\frac{\partial E\left(\alpha,I,\beta_{2}\right)}{\partial\beta_{2}} &= -2\int_{\Omega}\frac{D_{2}\left(\mathbf{x}\right)I\left(\mathbf{x}\right)C_{2}^{2}\left(\mathbf{x}\right)}{m^{2}I_{2}\left(\mathbf{x}\right)+\sigma^{2}}\,\mathrm{d}\mathbf{x}\,.
\end{aligned}$$

The estimated intensities I can be optimized for given α and β_2 directly to

$$\begin{aligned}
0 &= -2\sum_{i=1}^{2}\frac{\beta_{i}D_{i}\left(\mathbf{x}\right)C_{i}^{2}\left(\mathbf{x}\right)}{m^{2}I_{i}\left(\mathbf{x}\right)+\sigma^{2}} \\
\Longleftrightarrow\ 0 &= \frac{D_{1}\left(\mathbf{x}\right)C_{1}^{2}\left(\mathbf{x}\right)}{m^{2}I_{1}\left(\mathbf{x}\right)+\sigma^{2}}+\frac{\beta_{2}D_{2}\left(\mathbf{x}\right)C_{2}^{2}\left(\mathbf{x}\right)}{m^{2}I_{2}\left(\mathbf{x}\right)+\sigma^{2}} \\
\Longleftrightarrow\ 0 &= \left(I_{1}\left(\mathbf{x}\right)-I\left(\mathbf{x}\right)C_{1}^{2}\left(\mathbf{x}\right)\right)C_{1}^{2}\left(\mathbf{x}\right)\left(m^{2}I_{2}\left(\mathbf{x}\right)+\sigma^{2}\right) \\
&\quad +\beta_{2}\left(I_{2}\left(\mathbf{x}\right)-\beta_{2}I\left(\mathbf{x}\right)C_{2}^{2}\left(\mathbf{x}\right)\right)C_{2}^{2}\left(\mathbf{x}\right)\left(m^{2}I_{1}\left(\mathbf{x}\right)+\sigma^{2}\right) \\
\Longleftrightarrow\ 0 &= I_{1}\left(\mathbf{x}\right)C_{1}^{2}\left(\mathbf{x}\right)\left(m^{2}I_{2}\left(\mathbf{x}\right)+\sigma^{2}\right)-I\left(\mathbf{x}\right)C_{1}^{4}\left(\mathbf{x}\right)\left(m^{2}I_{2}\left(\mathbf{x}\right)+\sigma^{2}\right) \\
&\quad +\beta_{2}I_{2}\left(\mathbf{x}\right)C_{2}^{2}\left(\mathbf{x}\right)\left(m^{2}I_{1}\left(\mathbf{x}\right)+\sigma^{2}\right)-\beta_{2}^{2}I\left(\mathbf{x}\right)C_{2}^{4}\left(\mathbf{x}\right)\left(m^{2}I_{1}\left(\mathbf{x}\right)+\sigma^{2}\right) \\
\Longleftrightarrow\ 0 &= I_{1}\left(\mathbf{x}\right)C_{1}^{2}\left(\mathbf{x}\right)\left(m^{2}I_{2}\left(\mathbf{x}\right)+\sigma^{2}\right)+\beta_{2}I_{2}\left(\mathbf{x}\right)C_{2}^{2}\left(\mathbf{x}\right)\left(m^{2}I_{1}\left(\mathbf{x}\right)+\sigma^{2}\right) \\
&\quad -I\left(\mathbf{x}\right)\left(C_{1}^{4}\left(\mathbf{x}\right)\left(m^{2}I_{2}\left(\mathbf{x}\right)+\sigma^{2}\right)+\beta_{2}^{2}C_{2}^{4}\left(\mathbf{x}\right)\left(m^{2}I_{1}\left(\mathbf{x}\right)+\sigma^{2}\right)\right) \\
\Longleftrightarrow\ I\left(\mathbf{x}\right) &= \frac{I_{1}\left(\mathbf{x}\right)C_{1}^{2}\left(\mathbf{x}\right)\left(m^{2}I_{2}\left(\mathbf{x}\right)+\sigma^{2}\right)+\beta_{2}I_{2}\left(\mathbf{x}\right)C_{2}^{2}\left(\mathbf{x}\right)\left(m^{2}I_{1}\left(\mathbf{x}\right)+\sigma^{2}\right)}{C_{1}^{4}\left(\mathbf{x}\right)\left(m^{2}I_{2}\left(\mathbf{x}\right)+\sigma^{2}\right)+\beta_{2}^{2}C_{2}^{4}\left(\mathbf{x}\right)\left(m^{2}I_{1}\left(\mathbf{x}\right)+\sigma^{2}\right)}\,.
\end{aligned}$$

Given estimates for I and α the bleaching factor β_2 can also be explicitely optimized to

$$\begin{aligned}
0 &= -2\int_\Omega \frac{D_2(\mathbf{x})\, I(\mathbf{x})\, C_2^2(\mathbf{x})}{m^2 I_2(\mathbf{x})+\sigma^2}\,\mathrm{d}\mathbf{x}\\
\iff 0 &= -2\int_\Omega \frac{(I_2(\mathbf{x})-F_2(\mathbf{x}))\, I(\mathbf{x})\, C_2^2(\mathbf{x})}{m^2 I_2(\mathbf{x})+\sigma^2}\,\mathrm{d}\mathbf{x}\\
\iff 0 &= \int_\Omega \frac{I_2(\mathbf{x})\, I(\mathbf{x})\, C_2^2(\mathbf{x})}{m^2 I_2(\mathbf{x})+\sigma^2}\,\mathrm{d}\mathbf{x} - \beta_2\int_\Omega \frac{\left(I(\mathbf{x})\, C_2^2(\mathbf{x})\right)^2}{m^2 I_2(\mathbf{x})+\sigma^2}\,\mathrm{d}\mathbf{x}\\
\iff \beta_2 &= \frac{\int_\Omega \frac{I_2(\mathbf{x}) I(\mathbf{x}) C_2^2(\mathbf{x})}{m^2 I_2(\mathbf{x})+\sigma^2}\,\mathrm{d}\mathbf{x}}{\int_\Omega \frac{\left(I(\mathbf{x}) C_2^2(\mathbf{x})\right)^2}{m^2 I_2(\mathbf{x})+\sigma^2}\,\mathrm{d}\mathbf{x}}\,.
\end{aligned}$$

Numerical computation of the derivatives

The divergence in the derivative of the smoothness term has to be discretized in a stable way. For this we extend the discretization scheme proposed in [12] (Section 2.1.3) to 3-D. We define the 2nd order central differences of a discrete function $f:\mathbb{Z}^3\to\mathbb{R}$ as

$$\Delta_x^c f_{x,y,z} := \frac{f_{x+h_x,y,z}-f_{x-h_x,y,z}}{2h_x}\,,\qquad \Delta_y^c f_{x,y,z} := \frac{f_{x,y+h_y,z}-f_{x,y-h_y,z}}{2h_y}\,,\qquad \Delta_z^c f_{x,y,z} := \frac{f_{x,y,z+h_z}-f_{x,y,z-h_z}}{2h_z}\,,$$

the first order forward differences as

$$\Delta_x^f f_{x,y,z} := \frac{f_{x+h_x,y,z}-f_{x,y,z}}{h_x}\,,\qquad \Delta_y^f f_{x,y,z} := \frac{f_{x,y+h_y,z}-f_{x,y,z}}{h_y}\,,\qquad \Delta_z^f f_{x,y,z} := \frac{f_{x,y,z+h_z}-f_{x,y,z}}{h_z}\,,$$

and the first order backward differences as

$$\Delta_x^b f_{x,y,z} := \frac{f_{x,y,z}-f_{x-h_x,y,z}}{h_x}\,,\qquad \Delta_y^b f_{x,y,z} := \frac{f_{x,y,z}-f_{x,y-h_y,z}}{h_y}\,,\qquad \Delta_z^b f_{x,y,z} := \frac{f_{x,y,z}-f_{x,y,z-h_z}}{h_z}\,.$$

The gradient norms for the faces of the voxel at position $\mathbf{x}=(x,y,z)^\top$ are then given by

$$\begin{aligned}
\left\|\nabla\alpha_{x+\frac{h_x}{2},y,z}\right\| &\approx \sqrt{\left(\Delta_x^f\alpha_{x,y,z}\right)^2+\left(\frac{1}{2}\left(\Delta_y^c\alpha_{x+h_x,y,z}+\Delta_y^c\alpha_{x,y,z}\right)\right)^2+\left(\frac{1}{2}\left(\Delta_z^c\alpha_{x+h_x,y,z}+\Delta_z^c\alpha_{x,y,z}\right)\right)^2}\\
\left\|\nabla\alpha_{x-\frac{h_x}{2},y,z}\right\| &\approx \sqrt{\left(\Delta_x^b\alpha_{x,y,z}\right)^2+\left(\frac{1}{2}\left(\Delta_y^c\alpha_{x,y,z}+\Delta_y^c\alpha_{x-h_x,y,z}\right)\right)^2+\left(\frac{1}{2}\left(\Delta_z^c\alpha_{x,y,z}+\Delta_z^c\alpha_{x-h_x,y,z}\right)\right)^2}\\
\left\|\nabla\alpha_{x,y+\frac{h_y}{2},z}\right\| &\approx \sqrt{\left(\Delta_y^f\alpha_{x,y,z}\right)^2+\left(\frac{1}{2}\left(\Delta_x^c\alpha_{x,y+h_y,z}+\Delta_x^c\alpha_{x,y,z}\right)\right)^2+\left(\frac{1}{2}\left(\Delta_z^c\alpha_{x,y+h_y,z}+\Delta_z^c\alpha_{x,y,z}\right)\right)^2}\\
\left\|\nabla\alpha_{x,y-\frac{h_y}{2},z}\right\| &\approx \sqrt{\left(\Delta_y^b\alpha_{x,y,z}\right)^2+\left(\frac{1}{2}\left(\Delta_x^c\alpha_{x,y,z}+\Delta_x^c\alpha_{x,y-h_y,z}\right)\right)^2+\left(\frac{1}{2}\left(\Delta_z^c\alpha_{x,y,z}+\Delta_z^c\alpha_{x,y-h_y,z}\right)\right)^2}\\
\left\|\nabla\alpha_{x,y,z+\frac{h_z}{2}}\right\| &\approx \sqrt{\left(\Delta_z^f\alpha_{x,y,z}\right)^2+\left(\frac{1}{2}\left(\Delta_x^c\alpha_{x,y,z+h_z}+\Delta_x^c\alpha_{x,y,z}\right)\right)^2+\left(\frac{1}{2}\left(\Delta_y^c\alpha_{x,y,z+h_z}+\Delta_y^c\alpha_{x,y,z}\right)\right)^2}\\
\left\|\nabla\alpha_{x,y,z-\frac{h_z}{2}}\right\| &\approx \sqrt{\left(\Delta_z^b\alpha_{x,y,z}\right)^2+\left(\frac{1}{2}\left(\Delta_x^c\alpha_{x,y,z}+\Delta_x^c\alpha_{x,y,z-h_z}\right)\right)^2+\left(\frac{1}{2}\left(\Delta_y^c\alpha_{x,y,z}+\Delta_y^c\alpha_{x,y,z-h_z}\right)\right)^2}
\end{aligned}$$

and the divergence can be computed to

$$\begin{aligned}
\operatorname{div}\left(\psi'\left(\|\nabla\alpha_{x,y,z}\|^2\right)\nabla\alpha_{x,y,z}\right) &= \psi'\left(\left\|\nabla\alpha_{x+\frac{h_x}{2},y,z}\right\|^2\right)\Delta_x^f\alpha_{x,y,z} - \psi'\left(\left\|\nabla\alpha_{x-\frac{h_x}{2},y,z}\right\|^2\right)\Delta_x^b\alpha_{x,y,z} \\
&+ \psi'\left(\left\|\nabla\alpha_{x,y+\frac{h_y}{2},z}\right\|^2\right)\Delta_y^f\alpha_{x,y,z} - \psi'\left(\left\|\nabla\alpha_{x,y-\frac{h_y}{2},z}\right\|^2\right)\Delta_y^b\alpha_{x,y,z} \\
&+ \psi'\left(\left\|\nabla\alpha_{x,y,z+\frac{h_z}{2}}\right\|^2\right)\Delta_z^f\alpha_{x,y,z} - \psi'\left(\left\|\nabla\alpha_{x,y,z-\frac{h_z}{2}}\right\|^2\right)\Delta_z^b\alpha_{x,y,z}\,.
\end{aligned}$$

Dense Cone Integration

The employed numerical scheme of [7] uses a fast incremental integration strategy, that copes with the rather sparse ray sampling by incremental interpolation. This in the end captures most of the information contained in the attenuation field. However, it is a simplification of the real integration over infinitesimally thin rays and is a trade-off between numerical accuracy and computation time. We validated the fast integration scheme by comparing it to an alternative scheme in which we used one pixel thin rays. The number of rays was increased, so that even the widest cone diameter is still sufficiently sampled with respect to the processing resolution. Instead of the incremental interpolations, we first shear the attenuation field so that the integration direction is the z-direction. With this we only have to interpolate twice: once in the shearing transform and once to get the sub-pixel accurate cone origin in the sheared volume.

This alternative dense integration scheme with thin rays is still an approximation but should yield better reconstructions. However, due to the additional integration directions the approach is in practice not applicable with reasonable processing time.

We applied the attenuation correction with the dense integration scheme to a noise-free dataset to validate the energy formulation. The corresponding reconstruction is shown in supplementary figure S2 in the leftmost column. The original intensities and attenuation coefficients are qualitatively well reconstructed but the rmse is still high compared to the 380.7 reported in Figure 4b. There, the prior knowledge of sparse attenuations guides the optimization well into the right direction. When applying the prior to the dense integration scheme the best reconstruction can be achieved (Figure S2 3rd column). The second column shows the reconstruction using the proposed fast approximation without prior assumptions. Slight cross-shaped artifacts are visible which result from the not perfectly even cone sampling.

Fig S1 shows the course of the energy, the norm of the gradients projected onto the feasible direction, and the rmse of the intensities and attenuations. The dense ray model did not converge within 1000 iterations. The rmse of the intensities and the attenuations, however, changed only marginally after 200 iterations. The

sparsity prior yields higher energies that reach the same energy plateau after only 50 iterations. The strong changes in the rmse of both intensities and attenuations up to the 150th iteration show that a large range of possible solutions lead to similar energies. The fluctuations in the energy gradient are probably caused by frequent changes in the optimization direction. A reason for this are the inner fix-point iterations during the explicit intensity and beta update that lead to large steps in the search space. This, in turn, requires regular re-initialization of the second derivative approximation in the quasi-Newton solver.

In summary, these experiments show, that the minimization of the proposed energy is challenging and future work needs to focus on the optimization of the numerical approximations used.

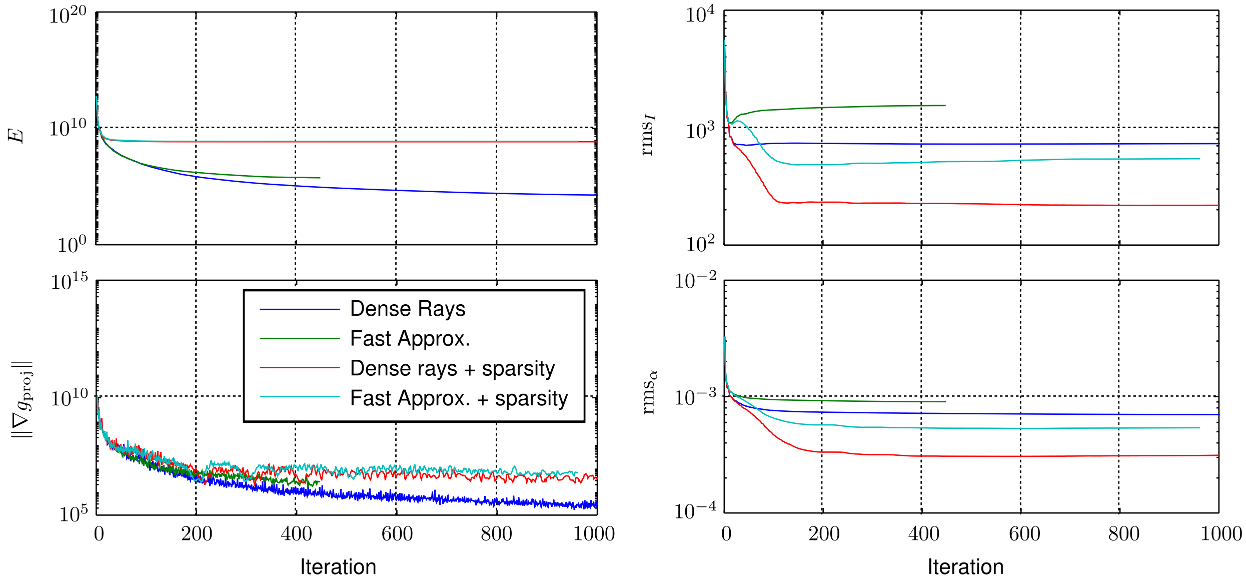

Figure S1: Effects of the integration scheme on (a) the energy, (b) the norm of energy gradient projected onto the closest feasible direction, (c) the rmse of the reconstructed intensities, and (d) the rmse of the reconstructed attenuations. "Dense Rays": Accurate cone integration using densely sampled thin rays; "Fast Approx": The proposed fast approximation using sparse sampling with cone-shaped rays; "sparsity": $\mu = 10^6$.

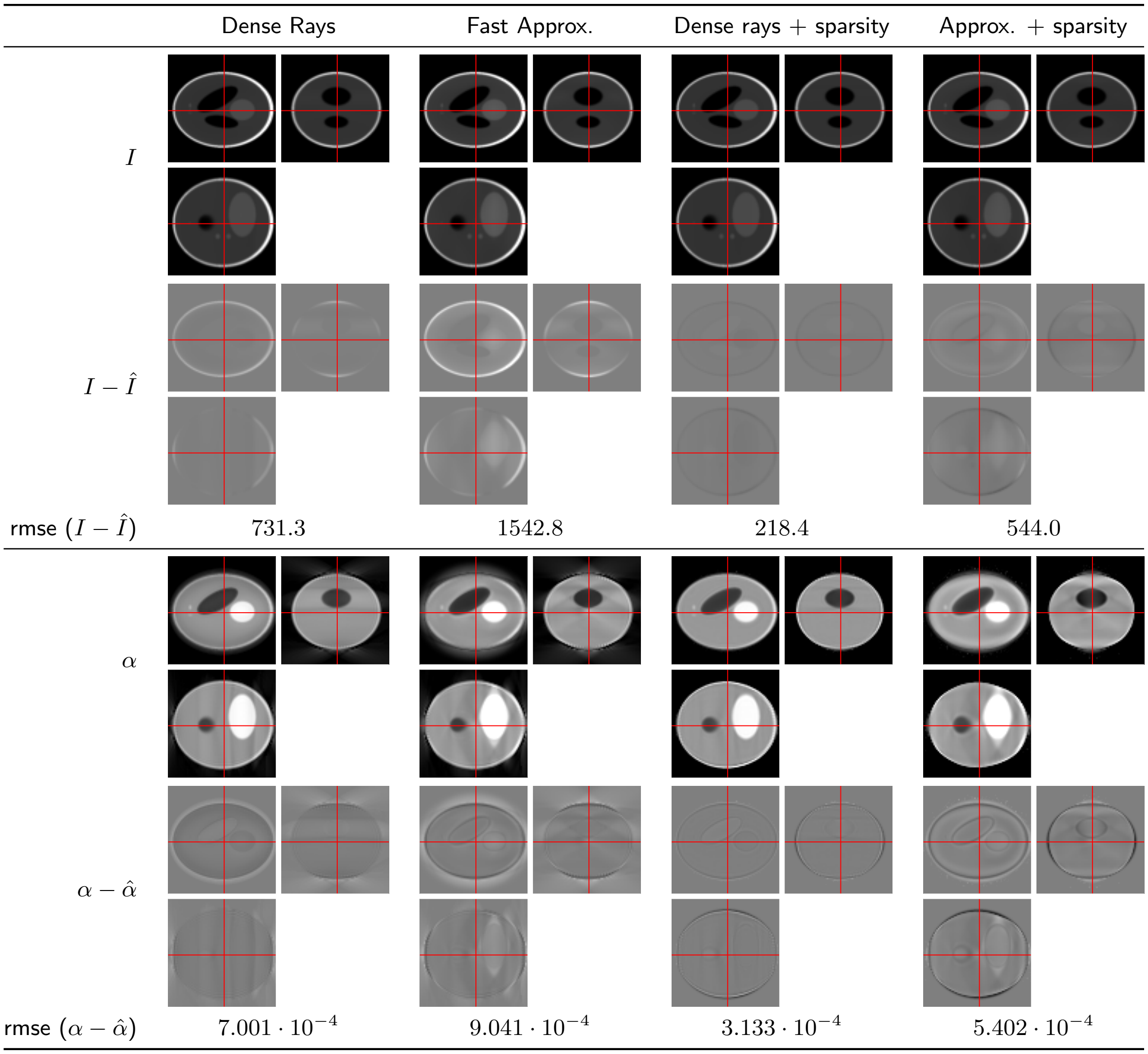

Figure S2: Effects of the integration scheme on the reconstruction of intensities and attenuations. Each column corresponds to one optimization setup, consisting of the indicated integration strategy. “Dense Rays”: Accurate cone integration using densely sampled thin rays; “Fast Approx”: The proposed fast approximation using sparse sampling with cone-shaped rays. “sparsity”: ($\mu = 10^6$).

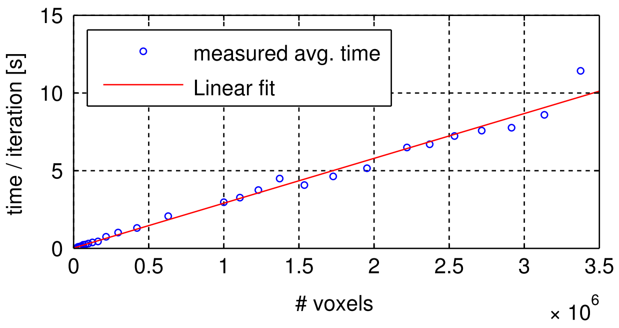

Figure S3: The effect of increasing volume sizes on the running time of the algorithm using the fast approximative cone integration scheme. Measurements (blue cricles) were performed on a workstation equipped with two Intel Xeon E5-2680 CPUs. The linear fit (red line) confirms the theoretical linear complexity of the algorithm in practice.
